# De Novo-Whole Genome Assembly of the Roborovski Dwarf Hamster (*Phodopus roborovskii*) Genome: An Animal Model for Severe/Critical COVID-19

**DOI:** 10.1093/gbe/evac100

**Published:** 2022-07-02

**Authors:** Sandro Andreotti, Janine Altmüller, Claudia Quedenau, Tatiana Borodina, Geraldine Nouailles, Luiz Gustavo Teixeira Alves, Markus Landthaler, Maximilian Bieniara, Jakob Trimpert, Emanuel Wyler

**Affiliations:** Department of Mathematics and Computer Science, Institute of Computer Science, Freie Universität Berlin, Takustr. 9, Berlin, Germany; Cologne Center for Genomics, University of Cologne, Cologne, Germany; Scientific Genomics Platforms, Max Delbrück Center for Molecular Medicine, Berlin, Germany; Scientific Genomics Platforms, Max Delbrück Center for Molecular Medicine, Berlin, Germany; Department of Infectious Diseases and Respiratory Medicine, Charité—Universitätsmedizin Berlin, Corporate Member of Freie Universität Berlin and Humboldt-Universität zu Berlin, Berlin, Germany; Max Delbrück Center for Molecular Medicine in the Helmholtz Association (MDC), Berlin Institute for Medical Systems Biology, Berlin, Germany; Max Delbrück Center for Molecular Medicine in the Helmholtz Association (MDC), Berlin Institute for Medical Systems Biology, Berlin, Germany; IRI Life Sciences, Institute of Biology, Humboldt-Universität zu Berlin, Berlin, Germany; Department of Mathematics and Computer Science, Institute of Computer Science, Freie Universität Berlin, Takustr. 9, Berlin, Germany; Institut für Virologie, Freie Universität Berlin, Berlin, Germany; Max Delbrück Center for Molecular Medicine in the Helmholtz Association (MDC), Berlin Institute for Medical Systems Biology, Berlin, Germany

**Keywords:** de novo, whole-genome assembly, Roborovski, dwarf, hamster, *Phodopus roborovskii*

## Abstract

The Roborovski dwarf hamster *Phodopus roborovskii* belongs to the *Phodopus* genus, one of the seven within Cricetinae subfamily. Like other rodents such as mice, rats, or ferrets, hamsters can be important animal models for a range of diseases. Whereas the Syrian hamster from the genus *Mesocricetus* is now widely used as a model for mild-to-moderate coronavirus disease 2019, Roborovski dwarf hamster shows a severe-to-lethal course of disease upon infection with the novel human coronavirus severe acute respiratory syndrome coronavirus 2.

**Significance**

The Roborovski dwarf hamster (*Phodopus roborovskii*) has become an important model for coronavirus disease 2019 research; however, research has been hampered due to the absence of a full genome annotation. We hereby present a draft genome assembly including gene annotation. This assembly can now serve as a key resource for many research projects employing this hamster species.

## Introduction

**Fig. 1.**
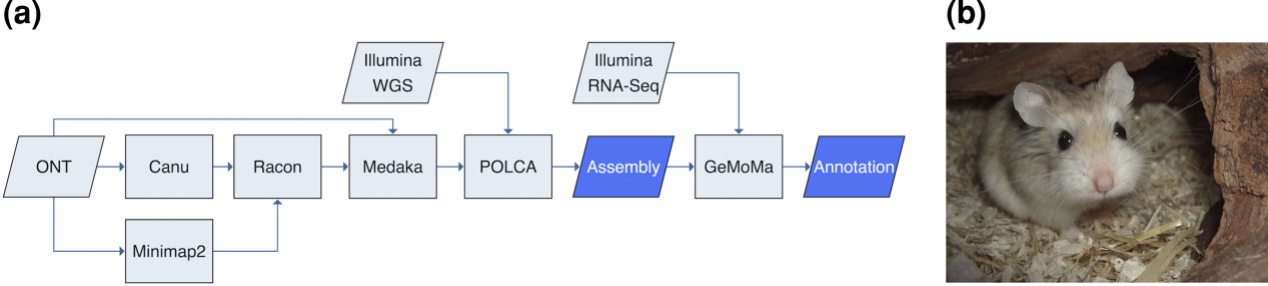
(*a*) Sketch of genome assembly and annotation pipeline and (*b*) photo of *Phodopus roborovskii*, male, 11 months old (source: wikipedia.org; author: Torben Schink, licensed under CC BY-SA 3.0).

The ongoing pandemic caused by the human severe acute respiratory syndrome coronavirus 2 (SARS-CoV-2) has made clear that traditional animal models such as mice and rats are not always suitable to study novel diseases and moreover, multiple animal models might be required to adequately reflect a variety of possible disease manifestations (Muñoz-Fontela et al. 2020). In fact, the importance of host factors has become strikingly evident in coronavirus disease 2019 (COVID-19), as the same virus causes disease severities that span from asymptomatic infections to severe acute respiratory distress syndrome and fatal multiorgan dysfunction (Guan, NEJM 2020). To solve immune mechanisms, identify putative targets of interventions and to test novel therapies and vaccination regimen animal models, ideally small animal models that reflect all presentations of COVID-19, are required (Lee and Lowen 2021; Veenhuis and Zeiss 2021). Nontransgenic mice and rats could not be productively infected with and consequently showed no weight loss or lung pathology in response to SARS-CoV-2 wildtype infection (Bao et al. 2020; Dinnon et al. 2020; Gu et al. 2020; Hassan et al. 2020; Muñoz-Fontela et al. 2020; Sun et al. 2020). In order to identify suitable models, species studied in the context of COVID-19 were chosen based on similarity in the SARS-CoV-2 receptor angiotensin converting enzyme-2 (ACE-2) predicted in silico (Pach et al. 2020; Wu et al. 2020; Devaux et al. 2021). COVID-19 research comprised nonhuman primates (Deng et al. 2020; Lu et al. 2020; Yu et al. 2020), cats (Shi et al. 2020), ferrets (Kim et al. 2020; Richard et al. 2020), and hamsters (Bertzbach et al. 2021) as animal models. Most small animals introduced to date show mild-to-moderate disease symptoms with resolving infections, including Syrian hamster (Chan et al. 2020; Imai et al. 2020; Kreye et al. 2020; Osterrieder et al. 2020; Sia et al. 2020). We previously introduced a dwarf hamster species, *Phodopus roborovskii* (fig. 1*b*), as a representative model for a severe course of disease including systemic immune activation and fatal disease outcome (Trimpert et al. 2020; Zhai et al. 2021). In our study, despite very similar ACE-2 sequences among the three analyzed *Phodopus* species, Roborovski dwarf hamsters (*P. roborovskii*), Campbell’s dwarf hamsters (*P. campbelli*), and Djungarian hamsters (*P. sungorus*), only the Roborovski dwarf hamster showed a severe disease manifestation following intranasal SARS-CoV-2 infection. The Roborovski dwarf hamster is, so far, the only nontransgenic animal that consistently develops severe disease and hyperinflammation of the lung following infection with SARS-CoV-2 (Muñoz-Fontela et al. 2020; Gantier 2021). Clinical signs develop within the first 48 h following infection and include drastic reduction in body temperature, substantial weight loss, forced breathing, ruffled fur, and lethargy. By histopathology, massive alveolar destruction and microthrombosis are evident in the lungs of infected animals, whereas other organs, including the brain, do not seem to be primarily involved in disease development. The rapid onset and fulminant course of pulmonary disease makes this species a valuable model to study severe courses of COVID-19 in humans and test therapies and vaccinations in the background of severe disease (Muñoz-Fontela et al. 2020; Gantier 2021). Before COVID-19, the preference for dietary unsaturated fatty acids and endocrine mechanisms was studied in *P. sungorus* (Hiebert et al. 2000; Scherbarth and Steinlechner 2010; Munley et al. 2018).

The natural habitat of *P. roborovskii* is the sandy deserts of the Mongolian Plateau, where they mainly eat seeds and insects. Within groups, amicable interactions are slightly more frequent than aggressive ones, corresponding to the overall more social interactions within *Phodopus* compared with, for example, *Mesocricetus* (golden hamster) species (Wilson et al. 2009). The Mongolian Plateau features a continental climate, with large seasonal climatic variation, the adaptation to which can be studied with endemic species such as *P. roborovskii* (Lv et al. 2016). This study on the population genetic structure employed the CytB gene sequence, in order to investigate the development of that species during the past 2–3 million years (Lv et al. 2016). *Phodopus* evolution was also studied using DNA repeat sequences from *P. roborovskii* and *P. sungorus*, which proposed that, due to the high abundance and diversity of such repeats, dwarf hamsters could be important models for evolutionary studies (Paço et al. 2015).

The novelty of this model however entails a problematic lack of reagents and tools to study immune reactions and other host factors. The absence of classical tools for molecular biology makes transcriptome and proteome analyses only more important as they may help to understand molecular reasons for severe COVID-19 and could supply information that helps finding reasonable medical interventions. Prerequisite for genomic and proteomic studies is a thoroughly annotated publicly available genome. As the closest annotated and available genome comes from a species in a different genus (*Mesocricetus auratus*, MesAur1.0), we describe here a scaffold-level genome assembly based on long- and short-read DNA sequencing, and annotated using RNA sequencing from heart and lung of *P. roborovskii* animals (fig. 1*a*).

## Results and Discussion

### Isolation and Sequencing of Genomic DNA

Genomic DNA was isolated from a whole-blood sample of an animal of about 7 weeks of age. From the same DNA sample, libraries were prepared for Promethion long-read sequencing and Illumina short-read sequencing.

### Assembly

The final assembly comprises a total of 2,078 (2,055 > 50 kb) contigs with a total length of 2.38 gb, an N50 of 25.78 mb, and an L50 of 30 (table 1 and supplementary table S1, Supplementary Material online). According to *QUAST*, 99.75% of 676.47 M paired-end short reads and 99.74% of 4.13 M long reads were mapped yielding average read depths of 80 and 34, respectively. The positive effect of genome assembly polishing using the described toolchain was confirmed by the genome completeness analysis with *BUSCO*. As the raw assembly as produced by *Canu* has a completeness of 79.3%, this value was improved to 85.9% with Racon, 88.8% with Medaka and finally reaches 92.0% after short-read polishing with *POLCA* (table 1 and supplementary table S2, Supplementary Material online). In the final step of the analysis, the screening with *Kraken2,* three contigs remained unclassified, three were classified as bacteria (total length: 314.68 kb), 74 as human, and the remaining 2028 matched either the golden hamster or mouse. Of the 74 contigs classified as human, 47 passed the final BLAST check and were included in the final cleaned assembly composed of 2,078 contigs.

**Table 1:** Assembly Statistics

Assembly statistics			
# contigs (≥0 bp)	2,078	BUSCO	
# contigs (≥25,000 bp)	2,075		
# contigs (≥50,000 bp)	2,055	Complete BUSCOs (C)	12,692
Total length (≥0 bp)	2,384,223,288	Complete and single-copy BUSCOs (S)	12,467
Total length (≥25,000 bp)	2,384,175,663	Complete and duplicated BUSCOs (D)	225
Total length (≥50,000 bp)	2,383,363,495	Fragmented BUSCOs (F)	267
N50	25,775,705	Missing BUSCOs (M)	839
L50	30	Total BUSCO groups searched	13,798

Key statistics of final genome assembly considering length, contiguity (left), and completeness based on BUSCO analysis (right). More detailed results are given in supplementary tables S1 and S2, Supplementary Material online.

### Annotation

Before quality and adapter trimming and filtering, the four RNA-Seq samples had between 31.2 and 13.5 million reads of which 96.4–98.1% passed the preprocessing stage and between 71.6% and 87.3% were uniquely mapped to the assembly with multimapping rates between 9.1% and 18.3% (supplementary table S3, Supplementary Material online). The final cleaned and curated annotation based on the prediction with the GeMoMa pipeline comprises 21,836 predicted transcripts in a total of 17,844 annotated gene loci.

## Materials and Methods

### Ethics Statement on Animal Husbandry

Roborovski dwarf hamsters were obtained through the German pet trade and housed in IVC units (Tecniplast). Hamsters were provided ad libidum with food and water and supplied with various enrichment materials (Carfil). For DNA extraction and sequencing, whole blood was obtained from uninfected control animals of a SARS-CoV-2 infection trial (Trimpert et al. 2020) that was performed according to all applicable regulations and approved by the relevant-state authority (Landesamt für Gesundheit und Soziales, Berlin, Approval Number 0086/20). RNA was extracted from SARS-CoV-2 infected and noninfected hamsters subject to an independent experiment (Trimpert et al. 2021) under the same permit. Briefly, anaesthetized hamsters were infected with 1 × 10^5^ focus forming units of SARS-CoV-2 (variant B.1, strain SARS-CoV-2/München-1.1/2020/929) in a 20-µl-cell culture medium. Animals were euthanized for sample collection on days 2 and 3 postinfection as previously described (Trimpert et al. 2020). Following the 3R principle, all materials used for this study were obtained from animals subject to independent animal experiments, no additional animals were used.

### Isolation of Genomic DNA

About 100 µl previously frozen whole blood was lysed by addition of 400 µl lysis solution CBV (Analytik Jena) and 10 µl proteinase K (20 mg/ml, Analytik Jena) followed by an incubation for 10 min ast 70 °C. Following this lysis step, another 10 µl proteinase K was added to perform an extended protein digestion for 30 min at 50 °C. DNA was extracted using a standard phenol/chloroform extraction with a first step of adding 1 ml liquefied TE saturated phenol (Carl Roth), gentle mixing by inverting the tube 20× and centrifugation at 10,000 × g for 10 min. The aqueous phase was aspirated with a cut pipette tip, mixed with 1 ml phenol/chloroform/isoamyl alcohol (25:24:1, Carl Roth) and mixed and centrifuged again as stated above. Again, the aqueous supernatant was carefully removed, mixed with 1 ml chloroform (Merck), and centrifuged for phase separation. The remaining aqueous phase was mixed with 1 ml absolute ethanol (Merck) and centrifuged for 30 min at 15,000 × g for DNA precipitation.

All steps were carried out with cut pipette tips and very gentle mixing to avoid shearing of the DNA.

### RNA Extraction

Lung pieces were stored in RNAlater (Thermo Fisher) for about 4 h before extraction. Afterwards, the tissue was lysed in a homogenizer (Eppendorf) in Trizol (ThermoFisher). For extraction of total RNA from whole blood, 250 µl anticoagulated (EDTA) sample was lysed by addition of 750 µl Trizol LS reagent (ThermoFisher). RNA was purified from Trizol using the Direct-zol RNA mini kit (Zymo Research) according to the manufacturer’s instructions.

### DNA Short-Read Sequencing

For short-read DNA sequencing, 1 µg of DNA was sonicated (Bioruptor, Diagenod), and the Illumina TruSeq DNA nano kit applied, using a slightly modified protocol with only one cycle of PCR to complete adapter structures. Following library validation and quantification (Agilent tape station, Peqlab KAPA Library Quantification Kit, and the Applied Biosystems 7900HT Sequence Detection System), sequencing was performed on an Illumina NovaSeq 6000 instrument with 2 × 150 paired-end sequencing.

### Long-Read Sequencing on PromethION

Sequencing libraries for long-read sequencing were prepared from 2.5 µg of unsheared genomic DNA, following the protocol of OxfordNanopore’s LSK109 kit (ONT, Oxford; https://store.nanoporetech.com/eu/media/wysiwyg/pdfs/SQK-LSK109/Genomic_DNA_by_Ligation_SQK-LSK109_-minion.pdf). DNA was end-repaired, A-tailed and purified (1 × of Ampure XP beads; Beckman Coulter). Then sequencing adapter with attached motor protein was ligated and the DNA was purified with 0.4× of Ampure beads. Quality and quantity of libraries were checked using HS gDNA 50 kb kit on fragment analyzer (Agilent) and dsDNA Qubit assay (ThermoFisher). Libraries were loaded three times, 30 fmol/30 fmol/14 fmol per 24 h, that is, 74 fmol in total. The complete runtime was 72 h.

### RNA Short-Read Sequencing

Poly(A)+ sequencing libraries were generated from total RNA using the NEBNext Ultra II Directional RNA Library Prep Kit (New England Biolabs) according to the manufacturer’s instruction, and sequenced on a NextSeq 500 device with single-end 76 cycles.

### De Novo Genome Assembly

Raw unprocessed reads were assembled using *Canu* assembler (Koren et al. 2017) with an estimated genome size of 2.1 gb and default parameters. This initial assembly was improved in a multistep procedure. As a first step, we mapped raw long reads using *minimap2* (Li 2018) and applied the *Racon* (Vaser et al. 2017) assembly polishing tool. In the next step, the *Racon*-polished genome was further improved with *Medaka* (https://nanoporetech.github.io/medaka) using again the raw long reads mapped with the *mini_align* script provided by the *Medaka* package, which also uses *minimap2*. For performance reasons, the polishing followed a recommendation in *Medaka’s* documentation and we first split the contigs into ten almost equally sized sets. Each set was processed using the subprogram *medaka consensus* and finally merged with *medaka stitch.* As a last polishing step, the result of *Medaka* was polished using the genomic short reads with *Polca* (Zimin and Salzberg 2020). Short reads were first trimmed and quality filtered using *bbduk* (https://sourceforge.net/projects/bbmap/) and filtered reads were used as input for *Polca*, which is based on *bwa-mem* read mapping and subsequent variant calling with *freebayes* (Garrison and Marth 2012). For evaluation of assembly quality and polishing effects, we applied the quality assessment tools *QUAST* (Mikheenko et al. 2018) and *BUSCO* (Simão et al. 2015) for estimation of genome completeness. At the final stage of the assembly process, we performed contamination screening of the polished assembly based on *Kraken2* (Wood et al. 2019) with database option *standard* augmented with genomes *M auratus* and *Mus musculus*, retaining those contigs classified either as one of these two species or unclassified. Contigs classified as bacteria were removed and those classified as human were further analyzed using *BLASTN* (Altschul et al. 1990) with database *nt* to eliminate possible contamination with human genetic material. For every contig, we summed up the bitscores per taxid for all hits with *e*-values below 1*e*−25 and assigned the species with the highest summed score. All contigs with hits to order *Rodentia* and/or without any hits passing the threshold remained in the final assembly. The versions and options for all tools in the bioinformatics toolchain are given in supplementary table S4, Supplementary Material online.

### Genome Annotation

RNA-Seq reads were quality trimmed and adapter sequences were removed with *Cutadapt* (Martin 2011) and filtered reads were mapped to the final polished assembly using the mapper *STAR* (Dobin et al. 2013). The mapped reads, together with closely related reference genomes and annotations of *M. musculus* (GRCm38.102), *R. norvegicus* (Rnor_6.0.102), and *M. auratus* (MesAur1.0.100)—obtained from ENSEMBL—were used a input for the hybrid genome annotation tool *GeMoMa* (Keilwagen et al. 2016, 2018) to predict gene loci. The mapped RNA-Seq reads were also used in a subsequent prediction of 5′ and 3′ UTRs. Finally, resulting gff files were converted to gtf format using *GffRead* (Pertea and Pertea 2020) and augmented with the original gene name(s) of the associated gene from the reference genomes with a custom Python script. Afterwards the annotation was cleaned according to the following scheme: If transcripts annotated for a single locus matched different gene names, only transcripts associated to the same gene name as the highest scoring (*GeMoMa* score) transcript for this locus were retained. In a second step, if the same gene name was associated with multiple annotated loci, only the locus with the higher top score was retained. In another postprocessing step, we removed exons shared by multiple genes as these fusions were artifacts introduced by GeMoMa’s UTR inference. As another filtering, transcripts with exons shorter than 15 bp or introns with noncanonical splice site consensus were eliminated. For transcript isoforms with identical exon boundaries, all but the one with the longest coding sequence were dropped using the script *agat_sp_fix_features_locations_duplicated.pl* from the AGAT toolkit (Dainat et al. 2021).

In another experiment based on a scRNA-Seq data set (accession number SRR17249853), we observed annotated 3′ UTRs were frequently too short. We extended them by a constant number of 1,000 bp whenever their distance to the next annotated feature (same or opposite strand) was at least 3,000 bp. This resulted in a 4% increase of assigned reads, 534 additionally detected genes, and ∼2500 genes with an increase of at least 20% assigned reads.

## Supplementary Material

Supplementary material is available at *Genome Biology and Evolution* online (http://www.gbe.oxfordjournals.org/).

## Supplementary Material

evac100_Supplementary_Data

## Data Availability

The genomic sequencing data underlying this article are available in the European Nucleotide Archive (ENA) and can be accessed with accession numbers ERR6740384, ERR6740385 (Illumina), and ERR6797440 (ONT). The accession numbers for the RNA-Seq raw reads are ERR6752847 (pr-d0-lung-1), ERR6752848 (pr-d2-lung-1), ERR6752849 (pr-d2-lung-2), and ERR6752850 (pr-d3-lung-2). The assembled genome together with annotation has been uploaded to figshare (https://doi.org/10.6084/m9.figshare.16695457), and submitted to ENA (GCA_943737965) using conversion tool EMBLmyGFF3 (Norling et al. 2018).
