# Supplementary material for: De Novo-Whole Genome Assembly of the Roborovski Dwarf Hamster (Phodopus roborovskii) Genome: An Animal Model for Severe/Critical COVID-19
Source: Genome Biol Evol. 2022 Jul 2;14(7):evac100. doi: 10.1093/gbe/evac100 (PMC9254642; doi:10.1093/gbe/evac100)
Supplement: evac100_Supplementary_Data [file evac100_supplementary_data.docx]

## Supplementary Material:

##### Table S1: QUAST results for assembly and after different polishing steps

| **Assembly** | **Canu** | **Canu + Racon** | **Canu + Racon + Medaka** | **Canu + Racon + Medaka + Polca** | **Canu +**  **Racon +**  **Medaka +**  **Polca +**  **Decont** |
| --- | --- | --- | --- | --- | --- |
| # contigs (>= 0 bp) | 2,112 | 2,109 | 2,108 | 2,108 | 2,078 |
| # contigs (>= 1000 bp) | 2,112 | 2,109 | 2,108 | 2,108 | 2,078 |
| # contigs (>= 5000 bp) | 2,109 | 2,108 | 2,108 | 2,108 | 2,078 |
| # contigs (>= 10000 bp) | 2,108 | 2,107 | 2,107 | 2,107 | 2,077 |
| # contigs (>= 25000 bp) | 2,106 | 2,105 | 2,105 | 2,105 | 2,075 |
| # contigs (>= 50000 bp) | 2,086 | 2,085 | 2,085 | 2,085 | 2,055 |
| Total length (>= 0 bp) | 2,381,232,474 | 2,386,917,682 | 2,388,794,081 | 2,388,036,258 | 2,384,223,288 |
| Total length (>= 1000 bp) | 2,381,232,474 | 2,386,917,682 | 2,388,794,081 | 2,388,036,258 | 2,384,223,288 |
| Total length (>= 5000 bp) | 2,381,220,014 | 2,386,913,209 | 2,388,794,081 | 2,388,036,258 | 2,384,223,288 |
| Total length (>= 10000 bp) | 2,381,210,169 | 2,386,903,358 | 2,388,788,685 | 2,388,030,857 | 2,384,217,887 |
| Total length (>= 25000 bp) | 2,381,168,154 | 2,386,861,164 | 2,388,746,456 | 2,387,988,633 | 2,384,175,663 |
| Total length (>= 50000 bp) | 2,380,358,702 | 2,386,050,351 | 2,387,934,087 | 2,387,176,465 | 2,383,363,495 |
| # contigs | 2,112 | 2,109 | 2,108 | 2,108 | 2,078 |
| Largest contig | 79,389,475 | 79,566,412 | 79,616,872 | 79,594,593 | 79,594,593 |
| Total length | 2,381,232,474 | 2,386,917,682 | 2,388,794,081 | 2,388,036,258 | 2,384,223,288 |
| GC (%) | 41.31 | 41.27 | 41.33 | 41.32 | 41 |
| N50 | 25,694,176 | 25,762,496 | 25,788,438 | 25,775,705 | 25,775,705 |
| N75 | 7,300,843 | 7,317,947 | 7,325,048 | 7,322,524 | 7,322,524 |
| L50 | 30 | 30 | 30 | 30 | 30 |
| L75 | 73 | 73 | 73 | 73 | 73 |
| Illumina reads | | | | | |
| # total reads | 1,354,820,496 | 1,354,379,523 | 1,354,069,760 | 1,354,067,462 | 1,354,038,936 |
| # left | 676,472,544 | 676,472,544 | 676,472,544 | 676,472,544 | 676,472,544 |
| # right | 676,472,544 | 676,472,544 | 676,472,544 | 676,472,544 | 676,472,544 |
| Mapped (%) | 99.73 | 99.75 | 99.75 | 99.75 | 99.75 |
| Properly paired (%) | 98.75 | 98.94 | 98.99 | 98.93 | 99.02 |
| Avg. coverage depth | 80 | 80 | 80 | 80 | 80 |
| Coverage >= 1x (%) | 99.66 | 99.57 | 98.94 | 98.96 | 98.99 |
| # N's per 100 kbp | 0 | 0 | 0 | 0 | 0 |
| ONT reads | | | | | |
| # total reads | 4,265,943 | 4,206,546 | 4,154,967 | 4,153,904 | 4,133,827 |
| Mapped (%) | 99.73 | 99.73 | 99.74 | 99.74 | 99.74 |
| Properly paired (%) | 0 | 0 | 0 | 0 | 0 |
| Avg. coverage depth | 34 | 34 | 34 | 34 | 34 |
| Coverage >= 1x (%) | 99.99 | 99.99 | 99.98 | 99.98 | 99.99 |
| # N's per 100 kbp | 0.00 | 0.00 | 0.00 | 0.00 | 0 |

##### Table S2: BUSCO results for assembly after different polishing steps. Decontamination of POLCA cleaned assembly did not affect BUSCO statistics.

| **Assembly** | **Canu** | **Canu + Racon** | **Canu + Racon + Medaka** | **Canu + Racon + Medaka + POLCA**  **(+ Decont)** |
| --- | --- | --- | --- | --- |
| Complete BUSCOs (C) | 10944 | 11857 | 12248 | 12692 |
| Complete and single-copy BUSCOs (S) | 10709 | 11616 | 12041 | 12467 |
| Complete and duplicated BUSCOs (D) | 235 | 241 | 207 | 225 |
| Fragmented BUSCOs (F) | 626 | 440 | 370 | 267 |
| Missing BUSCOs (M) | 2228 | 1501 | 1180 | 839 |
| Total BUSCO groups searched | 13798 | 13798 | 13798 | 13798 |

##### Table S3: Mapping statistics of RNA-Seq reads

| **Sample** | **pr-d0-lung-1** | **pr-d2-lung-1** | **pr-d2-lung-2** | **pr-d3-lung-2** |
| --- | --- | --- | --- | --- |
| Number of reads | 24,523,437 | 22,663,402 | 13,547,346 | 31,230,371 |
| Number of input reads (after quality filtering) | 23,667,474 | 22,230,068 | 13,222,122 | 30,107,224 |
| Average input read length | 74 | 74 | 74 | 74 |
| UNIQUE READS: | | | | |
| Uniquely mapped reads number | 18,414,197 | 18,347,689 | 11,543,772 | 21,555,625 |
| Uniquely mapped reads % | 77.80% | 82.54% | 87.31% | 71.60% |
| Average mapped length | 73.82 | 74.06 | 74.03 | 73.96 |
| Number of splices: Total | 2,714,011 | 3,019,367 | 1,750,395 | 3,522,768 |
| Number of splices: Annotated (sjdb) | 0 | 0 | 0 | 0 |
| Number of splices: GT/AG | 2,700,976 | 3,005,810 | 1,741,136 | 3,508,197 |
| Number of splices: GC/AG | 10,524 | 10,827 | 7,509 | 11,828 |
| Number of splices: AT/AC | 1,332 | 1,521 | 949 | 1,387 |
| Number of splices: Non-canonical | 1,179 | 1,209 | 801 | 1,356 |
| Mismatch rate per base, % | 0.17% | 0.15% | 0.15% | 0.16% |
| Deletion rate per base | 0.01% | 0.01% | 0.01% | 0.01% |
| Deletion average length | 1.51 | 1.48 | 1.51 | 1.48 |
| Insertion rate per base | 0.01% | 0.00% | 0.01% | 0.00% |
| Insertion average length | 1.39 | 1.35 | 1.36 | 1.35 |
| MULTI-MAPPING READS: | | | | |
| Number of reads mapped to multiple loci | 3,939,690 | 3,293,844 | 1,200,247 | 5,523,145 |
| % of reads mapped to multiple loci | 16.65% | 14.82% | 9.08% | 18.34% |
| Number of reads mapped to too many loci | 553,871 | 295,740 | 200,857 | 1,985,491 |
| % of reads mapped to too many loci | 2.34% | 1.33% | 1.52% | 6.59% |
| UNMAPPED READS: | | | | |
| Number of reads unmapped: too many mismatches | 0 | 0 | 0 | 0 |
| % of reads unmapped: too many mismatches | 0.00% | 0.00% | 0.00% | 0.00% |
| Number of reads unmapped: too short | 540,151 | 182,355 | 199,639 | 422,951 |
| % of reads unmapped: too short | 2.28% | 0.82% | 1.51% | 1.40% |
| Number of reads unmapped: other | 219,565 | 110,440 | 77,607 | 620,012 |
| % of reads unmapped: other | 0.93% | 0.50% | 0.59% | 2.06% |
| CHIMERIC READS: | | | | |
| Number of chimeric reads | 0 | 0 | 0 | 0 |
| % of chimeric reads | 0.00% | 0.00% | 0.00% | 0.00% |

#####

##### Table S4: Overview tools, versions and commands:

| **Tool** | **Version** | **Parameters** |
| --- | --- | --- |
| Assembly | | |
| Canu | 2.1.1 | genomeSize=2.1g -nanopore |
| Racon | 1.4.21, commit: b99f01f | racon_wrapper --split 300000000 -m 8 -x -6 -g -8 -w 500 |
| minimap2 | 2.17-r941 | -x map-ont |
| Medaka | 1.2.2 | Model: r941_prom_high_g4011; Analysis on 10 almost equally sized splits using: mini_align, medaka consensus, medaka stitch (all default parameters) |
| bbduk | 38.84 | qtrim=r trimq=20 trimpolyg=40 minlen=50 ref=adapters.fa ktrim=r k=23 mink=11 hdist=1 tbo |
| POLCA | Masurca 4.0.1 | default parameters |
| QUAST | 5.0.2 (bwa-mem 0.7.17) | --large --eukaryote (separately run for trimmed ILLUMINA and raw ONT reads) |
| BUSCO | 4.1.2 | -l glires_odb10 -m genome |
| Kraken2 | 2.1.1 | default parameters |
| blastn | 2.11.0+ | DB: nt, default parameters |
| Annotation | | |
| STAR | 2.7.5c | -outFilterType BySJout --outFilterMultimapNmax 20 --alignSJoverhangMin 8 --alignSJDBoverhangMin 1 --outFilterMismatchNmax 999 --outFilterMismatchNoverReadLmax 0.04 --alignIntronMin 20 --alignIntronMax 1000000 --alignMatesGapMax 1000000 |
| Cutadapt | 2.10 | -a AGATCGGAAGAGCACACGTCTGAACTCCAGTCA -a A{100} -g T{100} -q 20 -m 36 --length 75 |
| GeMoMa | 1.7.1 | GeMoMa-1.7.1.jar CLI GeMoMaPipeline threads=1 r=MAPPED ERE.s=FR_UNSTRANDED ERE.c=true AnnotationFinalizer.r=NO AnnotationFinalizer.u=YES GeMoMa.p=1 |
